# Combined Syngas and Hydrogen Production using Gas Switching Technology

**DOI:** 10.1021/acs.iecr.0c04335

**Published:** 2021-02-28

**Authors:** Ambrose Ugwu, Abdelghafour Zaabout, Felix Donat, Geert van Diest, Knuth Albertsen, Christoph Müller, Shahriar Amini

**Affiliations:** † Department of Energy and Process Engineering, Norwegian University of Science and Technology, Trondheim, 7491, Norway; ‡ Process Technology Department, SINTEF Industry, Trondheim, 7465, Norway; § Laboratory of Energy Science and Engineering, ETH Zürich, Zurich, 8092, Switzerland; ∥ Euro Support Advanced Materials B.V, Uden, 5405, The Netherlands; ⊥ Department of Mechanical Engineering, University of Alabama, Tuscaloosa, 35487, United States

## Abstract

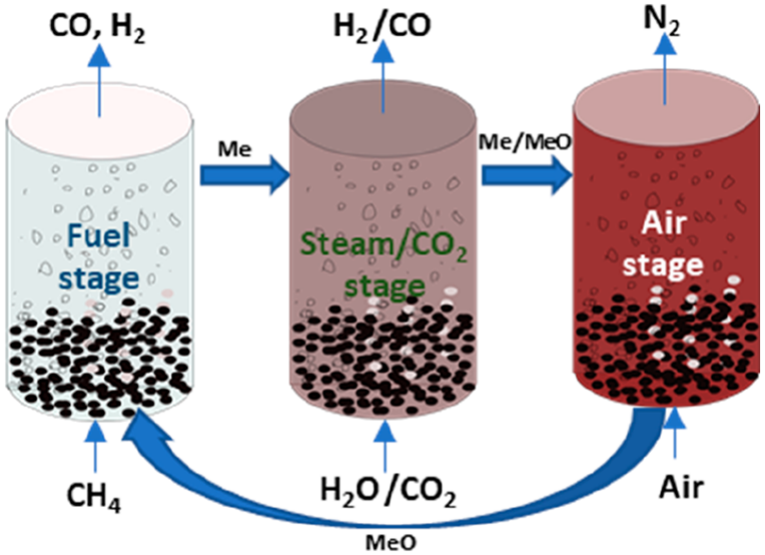

This paper focuses on the experimental demonstration of a three-stage GST (gas switching technology) process (fuel, steam/CO_2_, and air stages) for syngas production from methane in the fuel stage and H_2_/CO production in the steam/CO_2_ stage using a lanthanum-based oxygen carrier (La_0.85_Sr_0.15_Fe_0.95_Al_0.05_O_3_). Experiments were performed at temperatures between 750–950 °C and pressures up to 5 bar. The results show that the oxygen carrier exhibits high selectivity to oxidizing methane to syngas at the fuel stage with improved process performance with increasing temperature although carbon deposition could not be avoided. Co-feeding CO_2_ with CH_4_ at the fuel stage reduced carbon deposition significantly, thus reducing the syngas H_2_/CO molar ratio from 3.75 to 1 (at CO_2_/CH_4_ ratio of 1 at 950 °C and 1 bar). The reduced carbon deposition has maximized the purity of the H_2_ produced in the consecutive steam stage thus increasing the process attractiveness for the combined production of syngas and pure hydrogen. Interestingly, the cofeeding of CO_2_ with CH_4_ at the fuel stage showed a stable syngas production over 12 hours continuously and maintained the H_2_/CO ratio at almost unity, suggesting that the oxygen carrier was exposed to simultaneous partial oxidation of CH_4_ with the lattice oxygen which was restored instantly by the incoming CO_2_. Furthermore, the addition of steam to the fuel stage could tune up the H_2_/CO ratio beyond 3 without carbon deposition at H_2_O/CH_4_ ratio of 1 at 950 °C and 1 bar; making the syngas from gas switching partial oxidation suitable for different downstream processes, for example, gas-to-liquid processes. The process was also demonstrated at higher pressures with over 70% fuel conversion achieved at 5 bar and 950 °C.

## 1 Introduction

Natural gas is considered to be an important energy source in the decarbonization roadmap of fossil fuels considering its availability and low carbon footprint compared to other fossil fuels such as crude oil or coal.^1^ However, the direct utilization of natural gas is associated with CO_2_ emissions, thus shifting the focus toward its conversion to syngas (a mixture of hydrogen and carbon monoxide), hydrogen, and other valuable chemicals.^2^ Syngas can be produced from natural gas through six different ways:^3^ (i) Steam methane reforming (SMR), (ii) partial oxidation of methane (POX), (iii) dry methane reforming (DMR), (iv) combined methane reforming (CMR, a combination of SMR and DMR), (v) autothermal reforming (ATR, a combination of SMR and POX), and (vi) trireforming (TMR, a combination of SMR, DMR, and POX). However, only three (POX, SMR, and ATR) of the six technologies have been commercialized.^4,5^ Although SMR is commercialized, this technology is very energy-intensive and usually associated with high CO_2_ emissions. Partial oxidation of methane (POX) is more energy-efficient than SMR,^6^ but the conventional route (reaction R1) requires an air separation unit (ASU) for oxygen production, which increases the investment/capital costs and is also associated with CO_2_ emissions if nonrenewable electricity is used for powering the ASU. Nevertheless, POX remains an attractive technology when targeting its integration with gas-to-liquid (GTL) technologies for producing fuels, such as methanol or other higher hydrocarbons, because the produced syngas has a H_2_/CO ratio ranging between 1 and 2.^7−10^
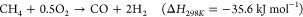
R1Chemical looping partial oxidation (CLPOX) of methane has been introduced to remove the need for the capital-intensive ASU by utilizing metal oxide-based oxygen carriers^11−14^ that can provide the oxygen for the partial oxidation reaction through circulation between two reactors, namely, the fuel and air reactors. The CLPOX of methane occurs through a heterogeneous reaction with the lattice oxygen of the oxygen carrier (reaction R3) in the fuel reactor. The oxygen carrier is then circulated to a second reactor, for the regeneration of its lattice oxygen with air in an exothermic reaction (reaction R12) that also supplies the required heat to the process (the partial oxidation reaction becomes endothermic when gaseous oxygen is substituted with lattice oxygen). This way CO_2_ emission is intrinsically avoided due to the inherently separated feed of air and CH_4_ to the two reactors. CLPOX shares similar advantages with the conventional chemical looping reforming (CLR), which has received increasing attention over the last two decades due to its prospects of increasing the process efficiency through heat integration.^15−18^ For material development, CLPOX exhibits an advantage over CLR in terms of cost and availability since metal oxides (oxygen carrier) are not required to be catalytically active for the hydrocarbon.^19−21^ CLPOX offers the flexibility to control the H_2_/CO ratio of the produced syngas by simply adjusting the process conditions, cofeeding CH_4_, H_2_O, and/or CO_2_ in the syngas production step.^22,23^

**Figure 1:**
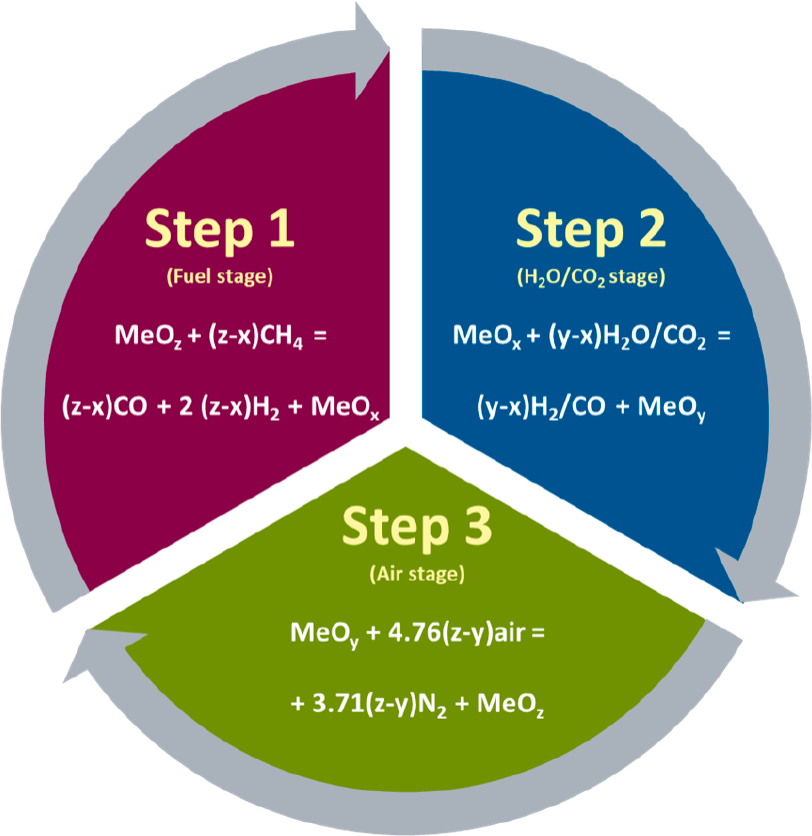
Redox cycle for the three-step chemical looping (CLPOX) process for the combined syngas and H_2_/CO production.

In this study, this technology has been extended to combine syngas and pure hydrogen production in a three-step process (CLPOX-H_2_) as illustrated in Figure 1. The three steps of the CLPOX-H_2_ are as follows: In the reduction step 1, the oxygen carrier is first reduced slightly when exposed to CH_4_ (reaction R2), thereby fully combusting the CH_4_. The oxygen carrier is then reduced further, but now the CH_4_ is partially oxidized by the lattice oxygen to produce syngas (reaction R3). In this step, CO_2_ and H_2_O could be utilized to control the syngas quality (i.e., H_2_/CO molar ratio). In the oxidation step 2, H_2_O/CO_2_ is fed to partially oxidize the oxygen carrier and produce H_2_/CO (reaction R9). In another oxidation step 3, the oxygen carrier is further oxidized by oxygen from the air for regeneration and production of heat (reaction R12). Step 3 could be avoided but that would reduce the overall heat generated from the process, thus requiring an additional external heat source to meet the heat requirement of the process.

**Figure 2:**
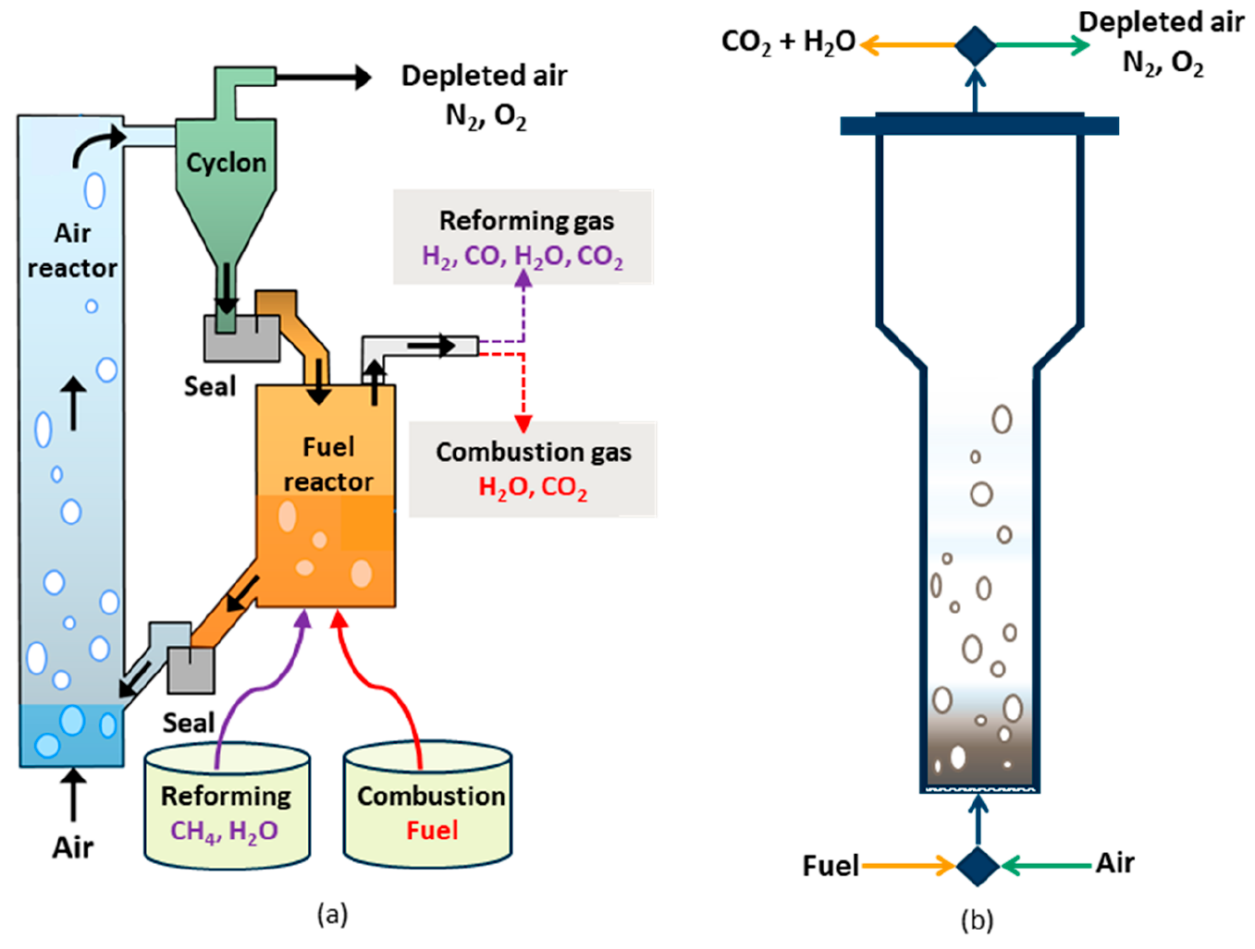
(a) Conventional chemical looping technology using CFB configuration;^44^ (b) gas switching technology proposed in this study.^45^

To maximize the economic and environmental benefits of CLPOX (or CLPOX-H_2_), a pressurized operation is required to improve the overall process efficiency and simplify its integration with downstream GTL processes. Chemical looping-based processes have been investigated at larger scales using interconnected circulating fluidized beds (CFB).^24,25^ Although the CFB configuration has been demonstrated at the lab^26−28^ and pilot^29−38^ scales for several chemical looping processes, pressurizing this configuration (Figure 2a) could be difficult for this application considering that each reactor needs to be pressurized individually with the need for precise control of the circulation of the oxygen carriers to fulfill the heat and mass balances of the process; only a few studies are reported on pressurized chemical looping using the interconnected fluidized bed configuration.^39^ The challenges magnify in three-steps processes such as CLPOX-H_2_, which would require three interconnected reactors with an oxygen carrier circulating between them. As a consequence, the studies on pressurized chemical looping operations are still very limited.^40−43^

**Figure 3:**
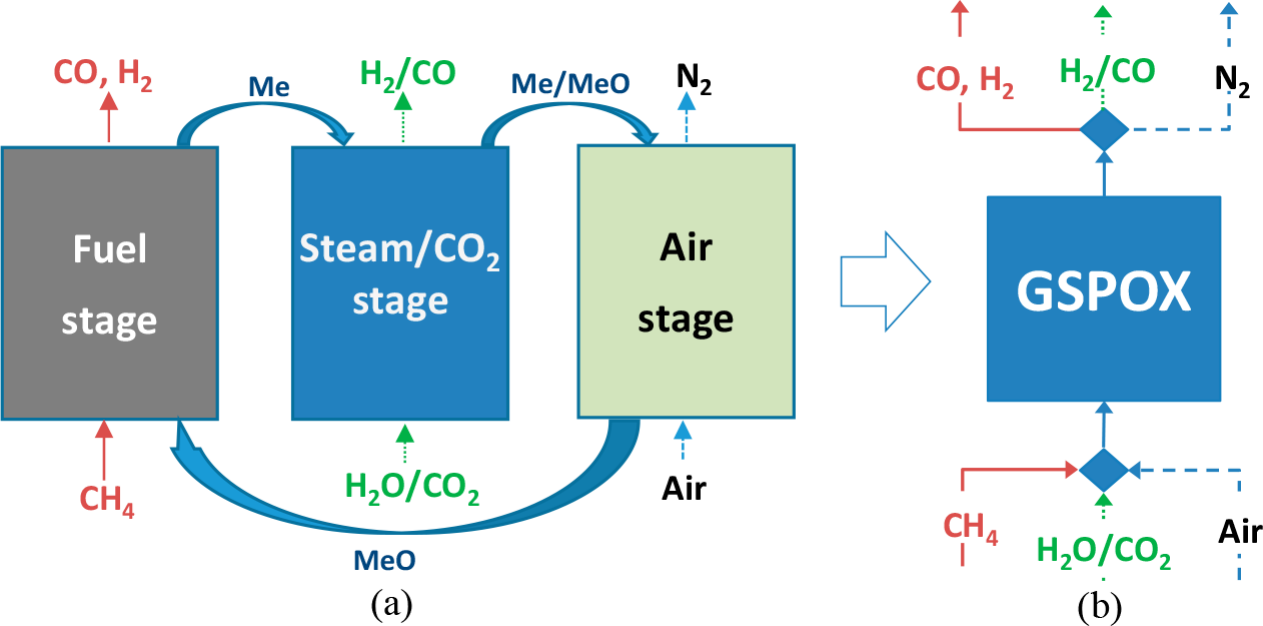
Three-stage chemical looping process for combined syngas production with integrated CO_2_/steam utilization to produce H_2_/CO: (a) conventional chemical looping arrangement; (b) the simplified gas switching technology under investigation.

Alternative reactor configurations have been proposed to address the need for pressurized operation. Among these alternatives, the gas switching technology (GST) reactor concept has been proven to be promising.^46−49^ The GST reactor concept utilizes a single fluidized bed vessel, in which gas feeds are alternated between the different reaction stages to oxidize and reduce the oxygen carrier (metal oxide) without requiring external solids circulation (Figure 2b). This greatly simplifies reactor operation and brings heat integration benefits as the reactions occur in one confinement as opposed to the traditional chemical looping concept that requires the circulation of oxygen carriers between separated reactors. The CLPOX process adopted is referred to as gas switching partial oxidation (GSPOX) and is illustrated in Figure 3.

Fuel Stage

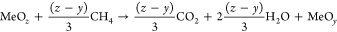
R2
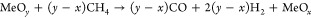
R3
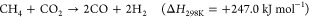
R4
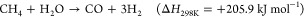
R5
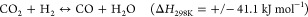
R6
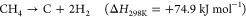
R7
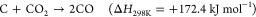
R8Steam/CO_2_ Stage

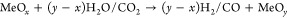
R9
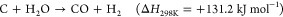
R10
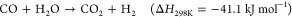
R11Air Stage

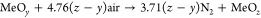
R12Note that *x*, *y*, and *z* represent the oxidation states of the oxygen carrier (*z* > *y* > *x*).

Like any other chemical looping-based process, the feasibility of GSPOX depends to a great extent on the oxygen carriers, which should be of low cost, and enable a high selectivity toward syngas production in the fuel stage and hydrogen production in the oxidation stage with steam.^50^ Perovskite-based metal oxides have demonstrated good performance for the production of syngas from CH_4_.^51−54^ Perovskites have the general formula of ABO_3_, where A represents a rare earth metal and/or an alkaline earth metal, and B is a transition metal.^55,56^ Perovskites generally possess good redox properties under the appropriate temperature and pressure conditions,^55,56^ offer more resistance to carbon deposition, and are thermodynamically suitable to convert CH_4_ to syngas.^10,57−59^ Perovskites have also been applied in the combined partial oxidation and H_2_O/CO_2_ splitting to produce syngas in the reduction step and H_2_/CO in the oxidation step.^60−63^

A La–Fe-based perovskite (La_0.85_Sr_0.15_Fe_0.95_Al_0.05_O_3_)_,_ that was developed, characterized, and tested at gram-scale, was upscaled to the kg-scale using spray drying in this study, and it was tested under real gas GSPOX conditions in a dense fluidized bed.^64,65^ A sensitivity study of this GSPOX process performance to the operating conditions such as CH_4_ molar concentration in the feed (8% was used in refs (64 and 65), which is far from real feed conditions of GSPOX), flow rate, operating temperature, and pressure was conducted to gain insight and understanding of the process behavior, and also to ascertain the best process conditions for the eventual scale-up of the process. A high focus was placed on demonstrating the tunability of the syngas composition delivered at the fuel stage to highlight the benefits of such a process in delivering custom designed syngas to different downstream GTL processes. Finally, a simultaneous redox reaction mechanism for coconversion CH_4_ and CO_2_ to syngas on this oxygen carrier (different from the conventional catalytic dry reforming) was experimentally demonstrated.

## 2 Experimental Demonstration

### 2.1 Oxygen Carrier

**Figure 4:**
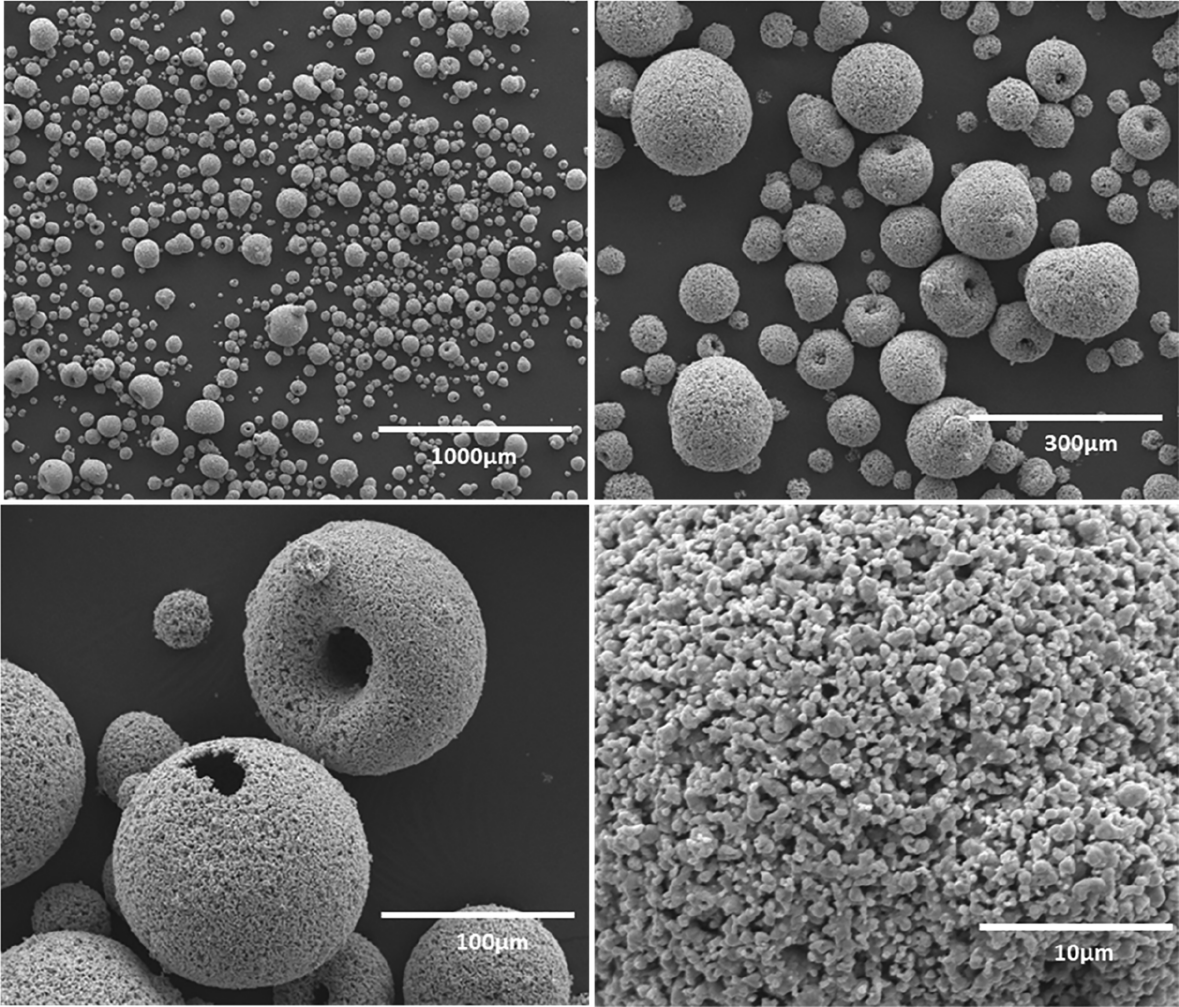
SEM image showing the particle distribution of the freshly synthesized La_0.85_Sr_0.15_Fe_0.95_Al_0.05_O_3_ oxygen carrier under investigation in this study.

**Figure 5:**
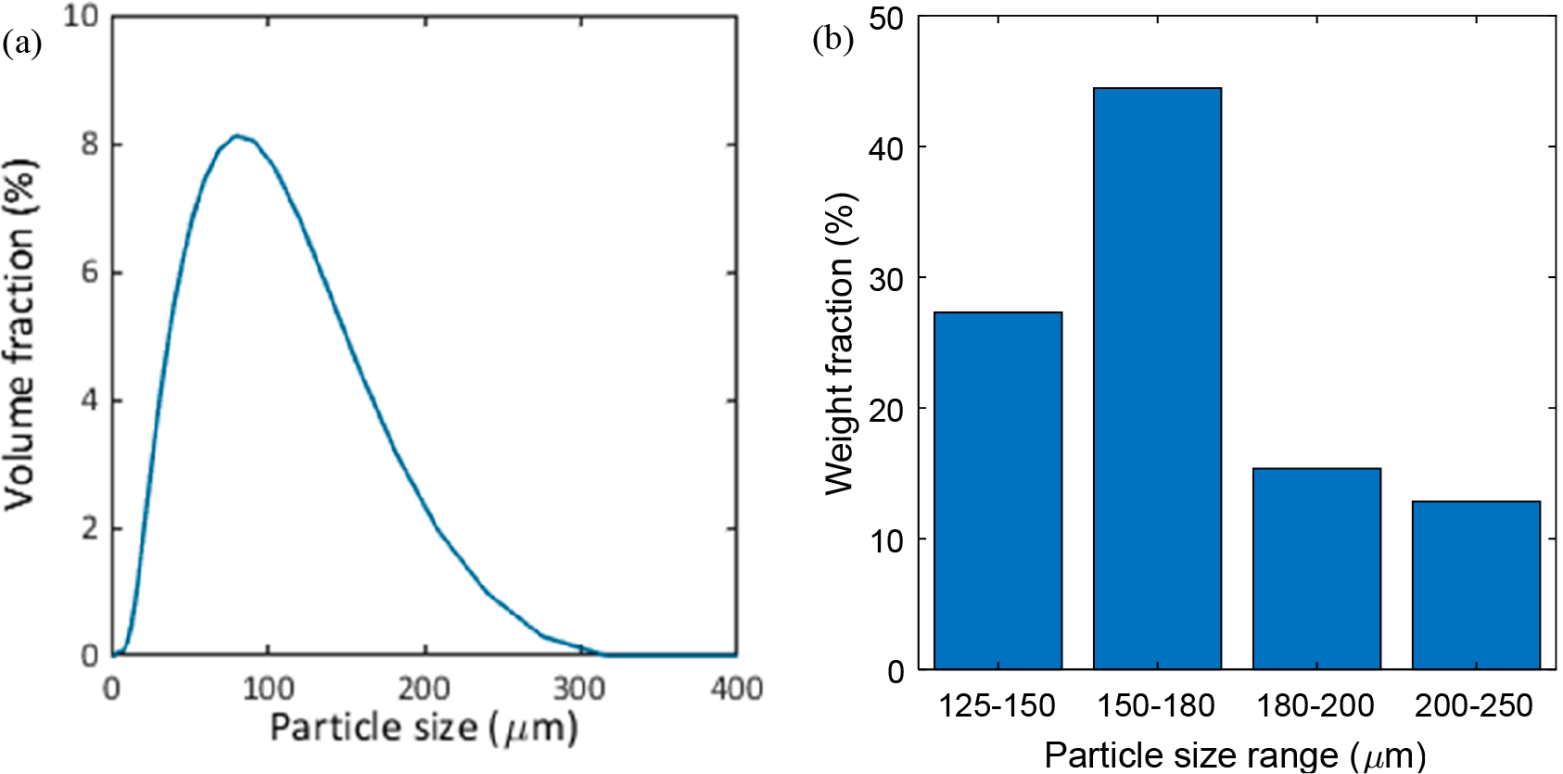
(a) Particle size distribution of calcined oxygen carrier spheres before sieving and (b) particle size distribution of material screened used in the reactor after sieving

**Figure 6:**
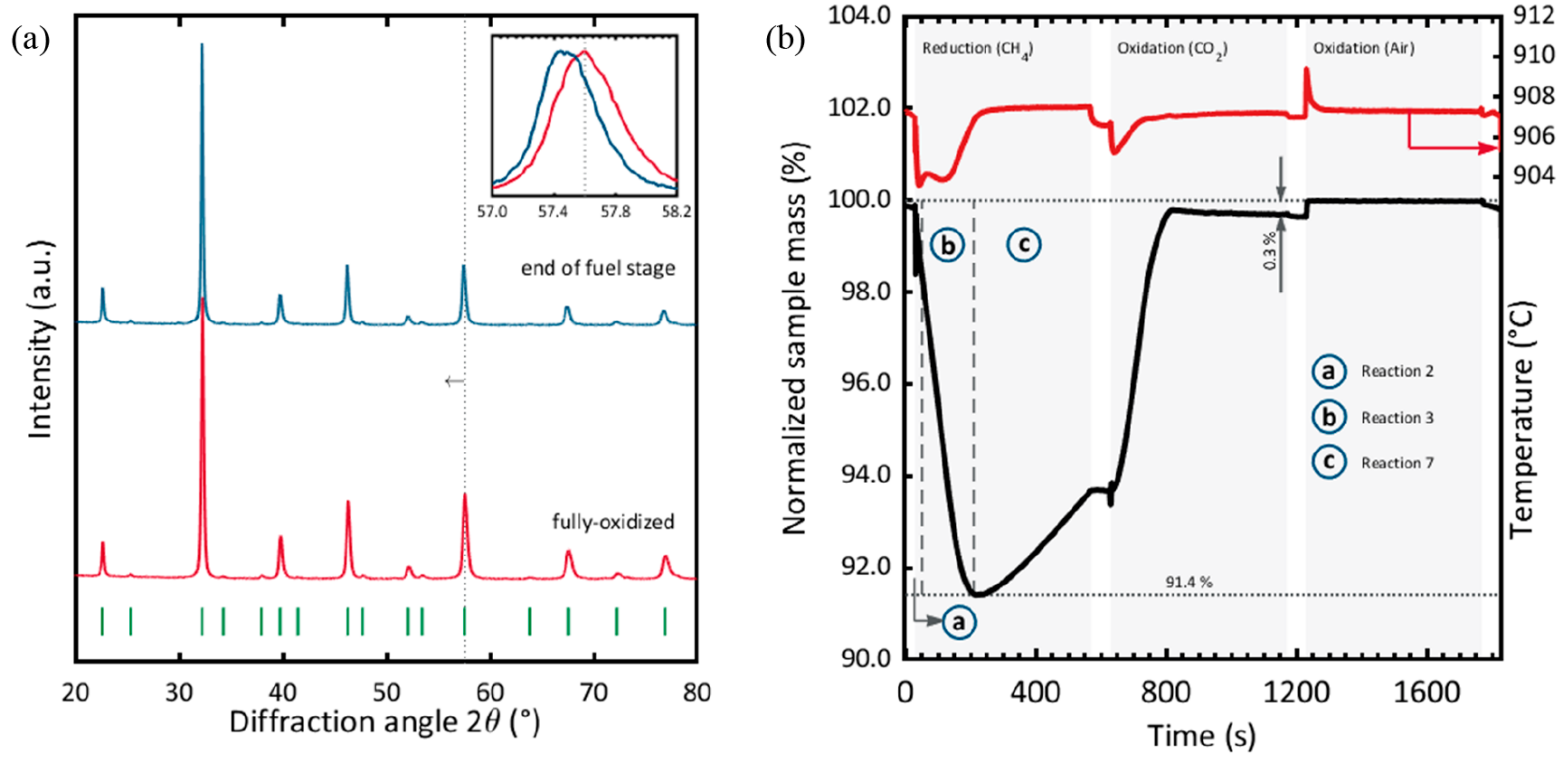
(a) XRD patterns of the synthesized oxygen carrier (La_0.85_Sr_0.15_Fe_0.95_Al_0.05_O_3-δ_) collected in the fully oxidized state and at the end the fuel stage (before the air oxidation step). The dotted vertical line indicates a shift in peak position, as can be seen more clearly in the inset. All indexed diffraction peaks correspond to the perovskite phase.^65^ (b) Normalized sample mass and temperature of the oxygen carrier measured in the TGA for different reducing and oxidizing gas atmospheres. The dashed vertical lines plotted in the reduction segment separate the reduction stage in segments where three different reactions were dominant: the total oxidation of CH_4_ (a, reaction **2**); the partial oxidation of CH_4_ (b, reaction R3); and the cracking of CH_4_ (c, reaction R7).^64^

The oxygen carrier used had the composition La_0.85_Sr_0.15_Fe_0.95_Al_0.05_O_3_ and was prepared from La_2_O_3_, SrCO_3_, Fe_2_O_3_, and Al_2_O_3_ (technical grades) by solid-state processing. This starting materials composition was determined in a previous study^64^ in which the different elements were mixed in the given ratio and then milled to the specific particle size (D10:0.263 μm, D50:0.620 μm, D90:1.355 μm, D99:2.1587 μm), followed by drying and calcination at 1250 °C for 4 h (5 °C/min increment, 25 °C/min decrement). Small samples of the prepared materials were characterized first by X-ray diffraction (XRD) to ensure a phase-pure perovskite had formed. Due to the relatively small scale of the material production (few kilograms), a small spray dryer was used resulting in less homogeneous particle size. Therefore, the material needed to be screened and sieved before the application in the fluidized bed reactor. Figure 4 shows the SEM image of the synthesized La_0.85_Sr_0.15_Fe_0.95_Al_0.05_O_3_ spheres produced by spray-drying. Initially, the PSD of the calcined spheres was quite wide (Figure 5a), but the samples used in the GST reactor were sieved between 137–225 μm for the experimental demonstration (Figure 5b). The particles were porous and had a relatively low density (bulk density ∼1900 kg/m^3^) compared to the heavy elements included. The oxygen carrier was phase-pure, as is evident from the diffractogram shown in Figure 6a. The maximum oxygen storage capacity was 9 wt % at 900 °C.^64^

### 2.2 Experimental Setup

**Figure 7:**
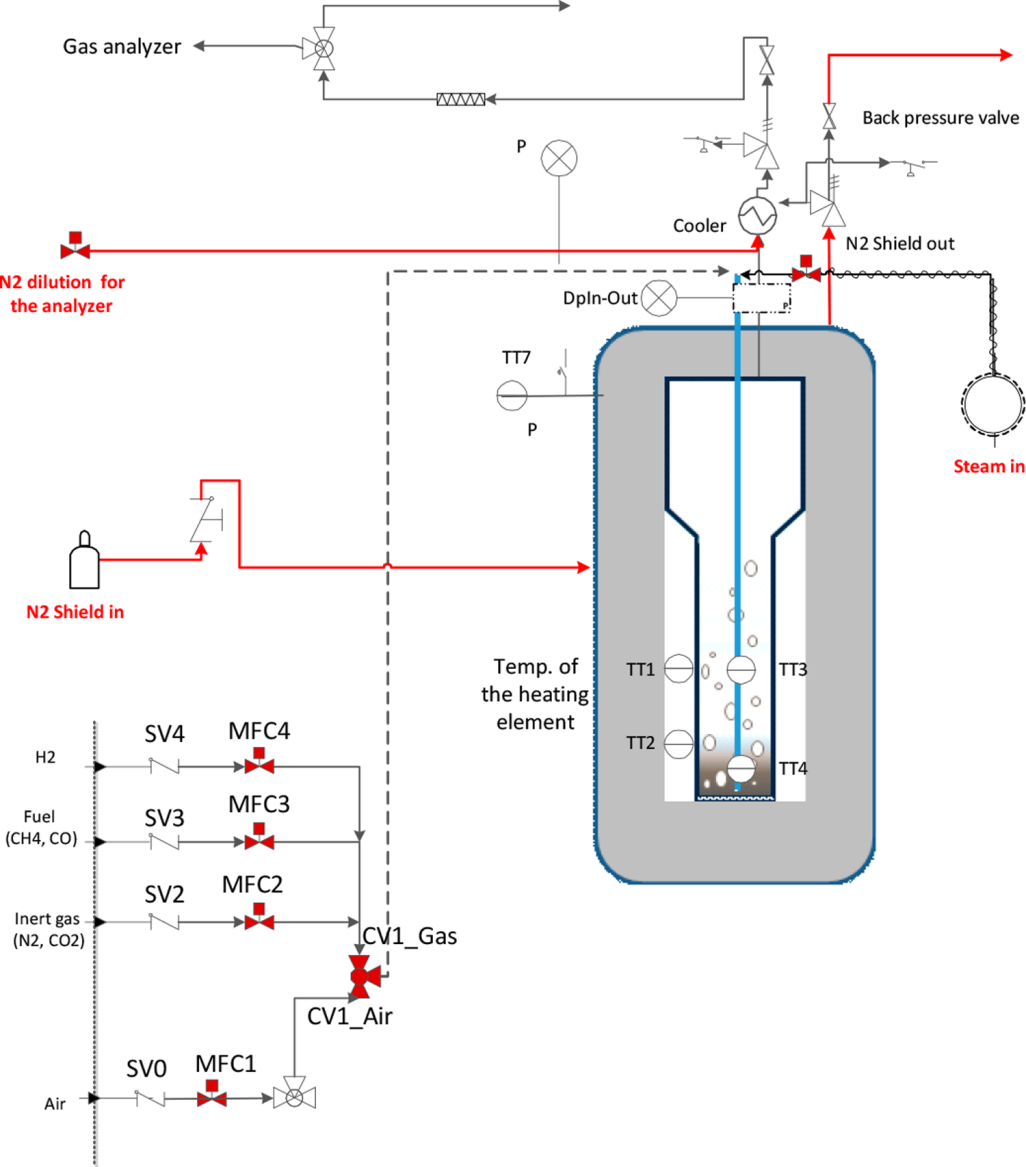
Experimental setup. SV04 represents stop valves and MFC1–4 represents mass flow controllers for air, the inert gas (N_2_ and CO_2_), the fuel (CH_4_, CO), and H_2_, respectively. TT1 and TT2 represent the temperature transmitter (thermocouple) that measures the temperature of the heating element on the reactor external circumference, while TT3 and TT4 represent temperature transmitters (thermocouple) that measure the bed temperature inside the reactor. P is pressure sensors while TT7 is the temperature transmitter (thermocouple) that measures the temperature inside the reactor shell.

The experimental set up consisted of a fluidized bed reactor, the gas switching reactor, with 5 cm inner diameter and 50 cm height with a freeboard region at the top (expanding from a 5 cm to a 10 cm diameter) to minimize particle entrainment (Figure 7). The total height of the reactor, including the body and the freeboard, was 90 cm. The reactor vessel was made of Inconel 600 to withstand high temperatures up to 1000 °C. Gas was fed into the reactor using a lance extending toward to bottom of the reactor. Heat was supplied to the reactor through an external electrical heating element wound around the reactor vessel and covered with a 25 cm thick insulation. The process parameters, data acquisition, and logging were controlled through a LabVIEW application. Bronkhorst mass flow controllers were used to measure and control the gas feed into the reactor. A three-way valve separated the air and fuel feeds during the redox process. The outlet gas stream was cooled down through the heat exchanger before it was sent to ventilation. Gas was sampled after the cooler and sent to a gas analyzer for measuring the gas composition. A syngas analyzer (model ETG MCA 100 SYN P) was used to measure the gas composition. The temperature was measured using two thermocouples located 2 and 20 cm from the bottom inside the reactor. The pressure was measured at different locations and used for monitoring reactor operation. A back-pressure valve was placed after the cooler and used for maintaining the target set pressure up to 5 bar. A thermogravimetric analyzer (Mettler Toledo TGA/DSC1) was used to investigate the amount of lattice oxygen that can be transferred to/from the oxygen carrier under different gas environments (CH_4_/CO_2_/air) at 900 °C.

### 2.3 Methodology

#### 2.3.1 GSPOX Operation

Lab-scale experiments were conducted using the La-based oxygen carrier described in section 2.1 and the experimental setup shown in (Figure 7). About 460 g of the oxygen carrier was placed inside the reactor, corresponding to a 0.3 m static bed height. The GSPOX cycle consists of three stages: fuel, steam, and air stage (Figure 3). The reactor was first heated up to the target temperature at a ramp rate of 5 °C/min, followed by approximately 30 short redox cycles (oxidation and reduction) for 1 h to enhance the activity of the oxygen carrier (“activation”). After activation, the actual GSPOX cycling experiments started with the fuel stage, where CH_4_ was fed. The net reaction at this stage is endothermic thus requiring heat addition to ensure that gas conversion does not decrease extensively across the stage. It is possible to cofeed CH_4_ with CO_2_ and/or H_2_O to control the syngas quality (i.e., H_2_/CO molar ratio) and carbon deposition. The steam stage proceeded the fuel stage to partially reoxidize the oxygen carrier while producing hydrogen and gasifying any deposited carbon from the fuel stage. Air was fed after the steam stage to ensure complete oxidation of the oxygen carrier and the generation of heat to drive the process. A known amount of inert N_2_ gas was fed across the fuel stage to quantify the amount of all the species formed or converted through carbon and hydrogen balances. There was also a purging step included between the redox stages to avoid the direct contact of the fuel and the oxidant. The total gas flow rate ranged between 1 and 50 nL/min in all stages. The gas flow rate was chosen to ensure that the bed was fluidized and the flow was maintained (*U*/*U*_mf_ ∼ 10) within the bubbling/turbulent regime to achieve good solid mixing/heat transfer across the bed. The experiments were performed by varying the CH_4_ molar ratio from 10–60%, temperatures from 750–950 °C, and reactor pressures from 1–5 bar. The reactor behavior, effect of temperature, pressure, CH_4_ molar fraction, flow rate, and CO_2_/H_2_O utilization were evaluated using reactor performance indicators described in section 2.3.2.

#### 2.3.2 Reactor Performance Indicators

Different performance indicators were defined to evaluate the GSPOX process and identify appropriate conditions to achieve the maximum conversion of CH_4_ to syngas (H_2_ and CO). Note that a known amount of inert gas (N_2_) was fed at the fuel and steam stages respectively to quantify the amount of other gaseous species using the mole fractions recorded in the gas analyzer (eq 1). It is desired to have maximal gas conversion in the fuel stage and H_2_O/CO_2_ conversion in the steam/CO_2_ stage. The CH_4_ conversion and the fuel stage and H_2_O conversion are defined in eq 2 and eq 3, respectively. It is important to tune the syngas H_2_/CO ratio (eq 4) to meet the requirements of the downstream process where the produced syngas could be utilized. Carbon deposition may occur in the fuel stage which is quantified as a percentage of the total converted carbon sources (CH_4_ and CO_2_) fed at the fuel stage that produced solid carbon (eq 5). As mentioned earlier in this section, a known amount of inert gas (N_2_) was fed at the fuel stage to quantify the amount of the unconverted CH_4_ and CO_2_ from the gas analyzer mole fractions (eq 1). The unconverted CH_4_ and CO_2_ are subtracted from the amount of CH_4_ and CO_2_ fed at the fuel stage to determine the converted CH_4_ and CO_2_. The amount of deposited carbon (*n*_C__,out_fuel_) was quantified through a carbon balance (eq 6). Deposited carbon is released at the steam and air stages in the form of CO and CO_2_, thus negatively affecting the purity of produced H_2_ in the steam stage. In the fuel stage, many competing reactions can occur; it is, therefore, important to quantify the selectivity to the different species formed. The CO selectivity (eq 7) at the fuel stage is affected by the deposited solid carbon and CO_2_ selectivity (eq 8). The H_2_O production from the total oxidation of the fuel and the reverse water gas shift (RWGS) reaction affects the H_2_ selectivity (eq 9) while the produced solid carbon, CO_2_, and H_2_O affect the overall syngas selectivity (eq 10).
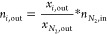
1
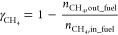
2
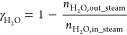
3
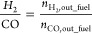
4
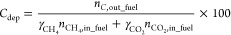
5
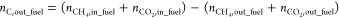
6
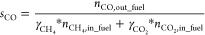
7
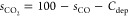
8
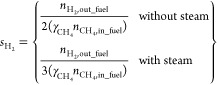
9
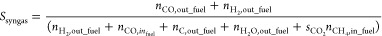
10

## 3 Result and Discussion

### 3.1 The GSPOX Process Behavior

**Figure 8:**
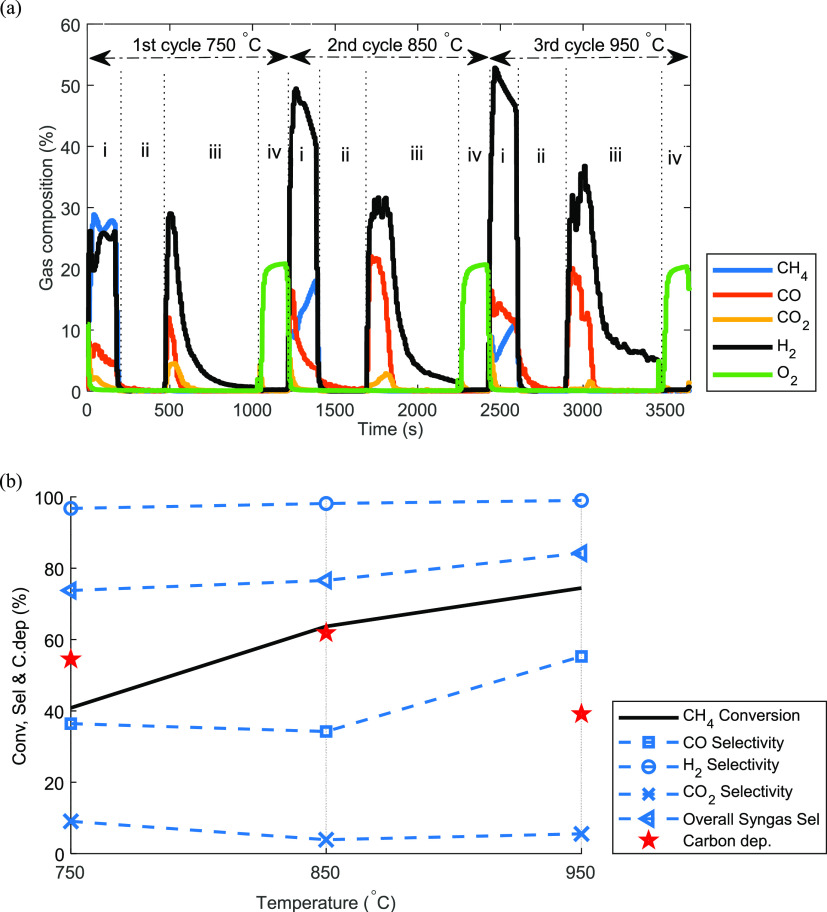
(a) Three cycles showing the transient gas composition under gas switching partial oxidation (GSPOX); (b) sensitivity of time-averaged values of key performance indicators in the fuel stage. CH_4_ molar fraction of 50% (diluted in N_2_) was kept constant at 1 bar, while the temperature was varied from 750 to 950 °C. Flow rates and time: (i) fuel stage (gas input: CH_4_, 4.1 nL/min; N_2_, 4.1 nL/min for 2.93 min); (ii) N_2_ purge (gas input: N_2_, 10 nL/min for 5 min); (iii) steam stage (gas input: H_2_O, 2 nL/min for 10 min); (iv) air stage (gas input: air 10 nL/min for 3 min).

Complete GSPOX cycles at atmospheric pressure and temperatures from 750 to 950 °C are shown in Figure 8a. The oxygen carrier reactivity was stable over the entire experimental campaign, with no signs of sintering/agglomeration observed despite being exposed to thermal stress and redox cycles. Sintering/agglomeration makes the particles fuse together, defluidize, thus making part of the bed to behave as a packed bed. With this, the particles will not be exposed equally to the reducing/oxidizing gases leading to hot spots, excessive reduction, and nonidentical gas composition over the cycles. On the other hand, identical gas composition over several cycles as observed during the experiment (Figure 8a) indicates that the mixing of the bed is good and there is no sign of sintering/agglomeration. The cycle starts with the fuel (reduction) stage where the oxygen carrier was exposed to CH_4_ (diluted with 50% N_2_). The overall reaction in the fuel stage is endothermic, unlike the conventional partial oxidation process using gaseous O_2_ feed. At the start of the fuel stage for the three temperatures, the CH_4_ was oxidized completely to CO_2_ and H_2_O, followed by a sharp transition toward partial oxidation with mostly syngas being produced. For this particular oxygen carrier composition, ∼4% of the redox-active lattice oxygen is selective for the total oxidation of CH_4_, whereas ∼96% of the redox-active lattice oxygen is selective for the partial oxidation of CH_4_, as can be seen from a control TGA experiment shown Figure 6b. During the reduction of the oxygen carrier, the perovskite phase transitions to La_2_O_3_, La_*x*_Sr_2-x_Fe_*y*_Al_1–*y*_O_4_, and metallic Fe in a single step.^64^ The high oxygen storage capacity of ∼9 wt % is associated with a change in the oxidation state of the iron component from Fe^3+^/Fe^4+^ to Fe^0^. Metallic Fe, that is, Fe^0^, catalyzes the decomposition of CH_4_ (reaction R7), which was apparent when the ratio of H_2_/CO measured in the off-gas increased above the theoretical value of 2. This is different from the results reported in previous studies using the La_0.6_Sr_0.4_Fe_0.8_Al_0.2_O_3-δ_ oxygen carrier with an oxide shell, that acts like a micromembrane via a thermochemical process,^66^ and La_1–x_Sr_*x*_FeO_3−δ_ via chemical looping^67^ with a H_2_O ratio ∼2, respectively, due to the different synthesis methods. However, the transient H_2_/CO ratio is similar to the first study with the same oxygen carrier in a gram-scale setup.^68^

At the steam stage, it can be seen that H_2_ was produced through the water-splitting reaction (reaction R9)—the partial oxidation of the oxygen carrier with steam. There was also gasification of the deposited carbon with steam (reaction R10) resulting in a large amount of CO produced in the first third of the steam stage. When all the deposited carbon was fully gasified, pure H_2_ production dominated the rest of the stage. In the oxidation stage with air, the rapid oxygen breakthrough suggests that most of the oxidation has been completed in the steam stage. As mentioned above, ∼96% of the redox-active lattice oxygen can be regenerated using mild oxidants such as H_2_O or CO_2_. It is worth noting that the observed rate of H_2_ production was about double the gaseous carbon products (CO and CO_2_) in the fuel stage, suggesting that both partial oxidation of the oxygen carrier and carbon gasification occurred simultaneously. A small amount of CO_2_ was also observed during the steam stage indicating the occurrence of the water gas shift (WGS) reaction (reaction R11), which decreased with temperature due to its exothermic nature. Finally, at the air stage, the still partially reduced oxygen carrier was regenerated completely. The reaction in this stage was highly exothermic generating part of the heat required to drive the endothermic reactions in the fuel stage to achieve autothermal operation.

Comparing the GSPOX behavior for the three operating temperatures tested (Figure 8a), it can be seen that the CH_4_ conversion almost doubled when the temperature was increased from 750 to 950 °C (Figure 8 b), indicating an improvement in the reaction kinetics as the temperature increases. The extent of carbon deposition also reduced with the increase in temperature (especially at 950 °C) in favor of an increased CO production (likely due to the increased oxygen release that simultaneously gasifies the depositing carbon), to a large extent contributing to an improved syngas selectivity. It was observed that the H_2_ selectivity improved when the temperature was increased from 750 to 850 °C and remained insensitive beyond 950 °C. This could be explained from Figure 8 showing that CO_2_ was produced simultaneously with syngas in the fuel stage indicating that the WGS reaction (reverse reaction R6) occurred in parallel with other reactions (reaction R3 to reaction R8) in the fuel stage. Recall that WGS reaction utilized H_2_O (which is the only competing product with H_2_) and CO to produce more H_2_ and CO_2_, thus increasing H_2_ selectivity. Since the WGS reaction (reverse reaction R6) is exothermic according to thermodynamics, the increase in temperature from 750 °C decreased the extent of the reaction until 950 °C where the reaction became negligible.

Despite the improvement in the degree of carbon deposition especially at 950 °C, the syngas H_2_/CO ratio remained above the expected value of 2 in the fuel stage (Figure 8a), and less than 80% H_2_ purity was achieved at the steam stage. It is worth mentioning that if syngas production is targeted, carbon deposition will not be an issue for this process as it is completely gasified within the subsequent steam stage, thus sustaining the oxygen carrier reactivity. Surprisingly, the carbon deposition reported in this study when less than 70% of the active lattice oxygen was consumed during the fuel stage was not observed in the gram-scale study with the same material,^64^ bringing into question a possible scale effect of the proposed gas switching technology as also reported in another study for H_2_ production through water splitting.^45^ It should, however, be noted that the gram-scale was performed with only 8% CH_4_ molar fraction as against 50% in the current study. The following section reports the results of a sensitivity study varying several operating parameters to evaluate their influence on key GSPOX process parameters.

### 3.2 Sensitivity Study

This section shows the effect of the operating and feed conditions on key performance indicators for the GSPOX process. Among others, a large focus is put on minimizing the carbon deposition (which also improves the purity of the produced hydrogen from the steam stage) and on showing the ability to tune the composition of produced syngas as a key feature of the process to respond to the feed specifications of the different downstream GTL processes.

#### 3.2.1 The Effect of CH_4_ Molar Fraction

**Figure 9:**
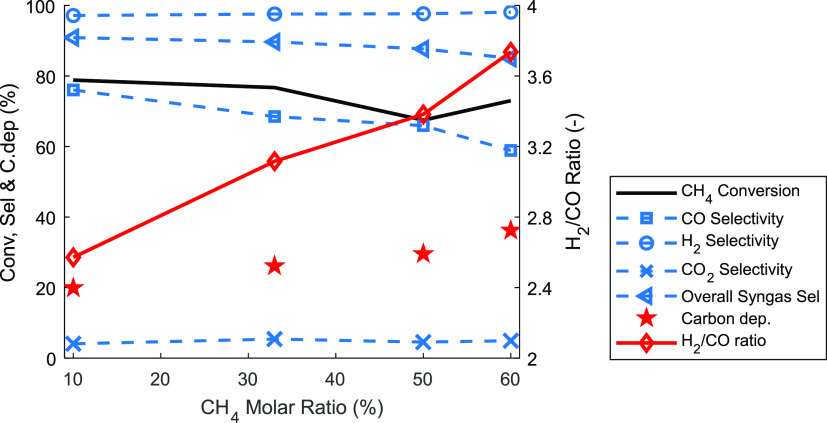
Sensitivity of key performance indicators to CH_4_ molar fraction at 1 bar operating pressure and 950 °C. (i) Fuel stage (gas input range: CH_4_, 0.6–3.72 nL/min; N_2_, 5.6–2.48 nL/min for 20–3.2 min); (ii) N_2_ purge (gas input: N_2_ 10 nL/min for 5 min); (iii) steam stage (gas input: H_2_O, 2 nL/min for 10 min); (iv) air stage (air, 10 nL/min for 3 min).

The effect of the CH_4_ molar fraction at the fuel stage was investigated under atmospheric conditions and 950 °C (Figure 9) while keeping the total gas flow rate constant. The time of the fuel stage was decreased proportionally with the CH_4_ molar fraction such that the total amount of CH_4_ fed during the fuel stage was kept constant. From the results shown in Figure 9, it can be seen that carbon deposition increased with the CH_4_ molar fraction. This finding further supports the GSPOX behavior explained in section 3.1, where it was shown that different active sites determine the dominant reactions/output of the GSPOX process. Although the fuel stage always started with a fully oxidized oxygen carrier, it is likely that the increased CH_4_ concentration in the reducing gas increased the rate of carbon deposition by locally reducing the oxygen carrier faster than expected. This increased carbon deposition reduced the CO selectivity, which in turn led to an increase of the H_2_/CO ratio to ∼3.7 when the CH_4_ molar fraction was 60%. Consequently, CH_4_ conversion was marginally affected by the carbon deposition. By inspecting Figure 8a, it can be seen that in the fuel stage the CH_4_ conversion decreased with time accompanied by a decrease in CO generation—a sign of increased carbon deposition which blocks the pores and limits the diffusion of gas into the active surface of the metal oxide.

Interestingly, the total amount of CO_2_ produced during the fuel stage was insensitive to the CH_4_ molar fraction, implying that the oxygen carrier was reduced to the same extent. This also confirms that the reduction of the oxygen carrier in the fuel stage occurred in two principal steps, in which the first short step involved the complete methane combustion to produce CO_2_ and H_2_O, while the second step involved the partial oxidation of methane after a certain amount of lattice oxygen had been removed from the oxygen carrier (850 and 950 °C in Figure 8a illustrate this behavior). From our previous work, the transition from the total to the partial oxidation of CH_4_ occurred when ∼3%–4% of the redox-active lattice oxygen was removed from the oxygen carrier, which can be seen also in Figure 6b.^64^ The H_2_/CO ratio of the syngas increased with carbon deposition indicating that the mechanism of carbon deposition is mainly methane cracking (reaction R7). The absence of CO_2_ and H_2_O in the second step (i.e., the partial oxidation) reduced the extent of side reactions, thus making H_2_ selectivity insensitive toward CH_4_ molar fraction. Despite that, the H_2_ selectivity remained unaffected, and the syngas selectivity decreased following the decrease in CO selectivity due to carbon deposition.

#### 3.2.2 The Effect of Flow Rate

**Figure 10:**
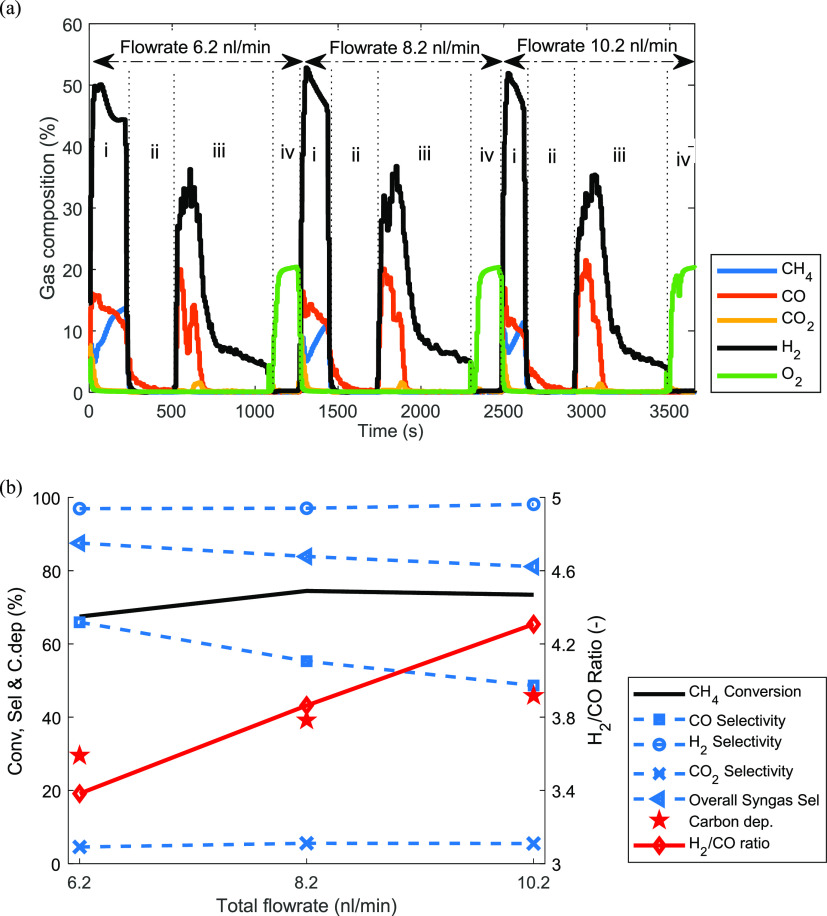
(a) The transient gas composition for different flow rates and (b) sensitivity of key performance indicators up to 50% CH_4_ molar fraction, 1 bar, and 950 °C. (i) Fuel stage (gas input: CH_4_, 3.1–5.1 nL/min; N_2_, 3.1–5.1 nL/min for 3.87–2.35 min); (ii) N_2_ purge (gas input: N_2_, 10 nL/min for 5 min; (iii) steam stage (H_2_O, 2 nL/min for 10 min); (iv) air stage (gas input: air, 10 nL/min for 3 min).

The effect of flow rate was investigated at 50% CH_4_ molar fraction (50% dilution with N_2_), 950 °C, and 1 bar by varying the flow rate between 6.2 nL/min and 10.2 nL/min (Figure 10). This flow rate range was selected to ensure that the reacting bed was always kept within the bubbling/turbulent fluidization regime. Similar to that in section 3.2.1, the total amount of CH_4_ fed during the fuel stage was kept the same by proportionally decreasing the stage time with the gas flow rate. The transient gas composition (Figure 10a) shows that the cycles for the three tested flow rates were almost identical, implying that the reactions involved in the three stages were fast enough to be independent of the gas residence time in the bed. This also suggests that the gas–solid contact was good in the studied range of the gas flow rates and that slippage of the reactant gases through the bed was avoided. Carbon deposition was apparent for the three cases as can be seen by the released CO and CO_2_ in the steam stage (after the fuel stage) marking the gasification of deposited carbon. H_2_ production through the partial oxidation of the oxygen carrier by steam was visible for the three tested cases. It can be clearly seen that the H_2_ concentration was around twice that of CO when carbon gasification occurred, while pure hydrogen was produced for the rest of the steam stage after all the carbon had been gasified.

From the time-averaged values shown in Figure 10b, the CH_4_ conversion increased slightly when the flow rate was increased from 6.2 to 8.2 nL/min, but it then remained relatively constant with a further increase. The improvement in CH_4_ conversion is a sign of improved mixing/gas–solids contact that counteracted the possible negative effect of reduced residence time. As expected, such improvement in the mixing of the gas and the particles would reduce bed segregation, prevent some of the solids to form a packed bed, ensure that the oxygen carrier is reduced uniformly in the entire bed, and reduce carbon deposition. However, Figure 10 contrarily shows that with increasing flow rate the carbon deposition increased. This may be as a result of the increased rate of reduction at higher flow rates which enhances carbon deposition (type 3 active site of Mihai et al.^69^) as described in section 3.1. Consequently, the CO selectivity increased with decreasing carbon deposition and the absence of the RWGS reaction (reaction R6). Interestingly, the selectivity to CO_2_ remained constant confirming that the oxygen carrier achieved the same level of reduction as described in section 3.2.1. As also explained in section 3.2.1, the H_2_ selectivity was also insensitive to the change in flow rate due to the good distinctive behavior of the two substeps of the fuel stage.

#### 3.2.3 The Effect of CO_2_ and H_2_O Utilization

**Figure 11:**
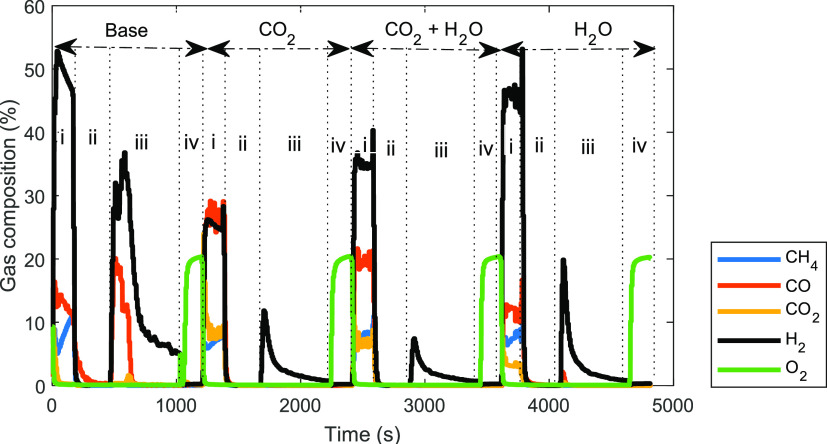
Transient gas composition for the base case without H_2_O/CO_2_ addition and other cases with H_2_O/CO_2_ addition as indicated in the plot at 50% CH_4_ molar fraction, 950 °C, and 1 bar. (i) Fuel stage, base case (gas input: CH_4_, 4.1 nL/min; N_2_, 4.1 nL/min for 2.93 min); CO_2_ case (gas input: CH_4_, 4.1 nL/min; CO_2_, 4.1 nL/min for 2.93 min); CO_2_+H_2_O case (gas input: CH_4_, 4.1 nL/min; CO_2_, 2.05 nL/min; H_2_O, 2.05 nL/min for 2.93 min); H_2_O case (gas input: CH_4_, 4.1 nL/min; H_2_O, 4.1 nL/min for 2.93 min); (ii) N_2_ purge (gas input: N_2_, 10 nL/min for 5 min); (iii) steam stage (gas input: H_2_O, 2 nL/min for 10 min); (iv) air stage (gas input: air, 10 nL/min for 3 min).

In an attempt to reduce carbon deposition and control the syngas quality, CO_2_ and H_2_O were cofed during the fuel stage. Four cases were investigated at atmospheric condition, 50% CH_4_ molar fraction and a temperature of 950 °C as follows: (i) base case, without CO_2_ and H_2_O addition (50% N_2_ and 50% CH_4_ molar fraction at fuel stage); (ii) CO_2_ case (50% CO_2_ and 50% CH_4_ molar fractions at fuel stage); (iii) H_2_O case (50% H_2_O and 50% CH_4_ at fuel stage); and (iv) CO_2_ + H_2_O case (25% CO_2_, 25% H_2_O, and 50% CH_4_ at fuel stage). The transient gas composition of the four cases (Figure 11) shows that the use of CO_2_ and H_2_O had a positive influence on the extent of carbon deposition, gas feed conversion, and syngas quality.

**Figure 12:**
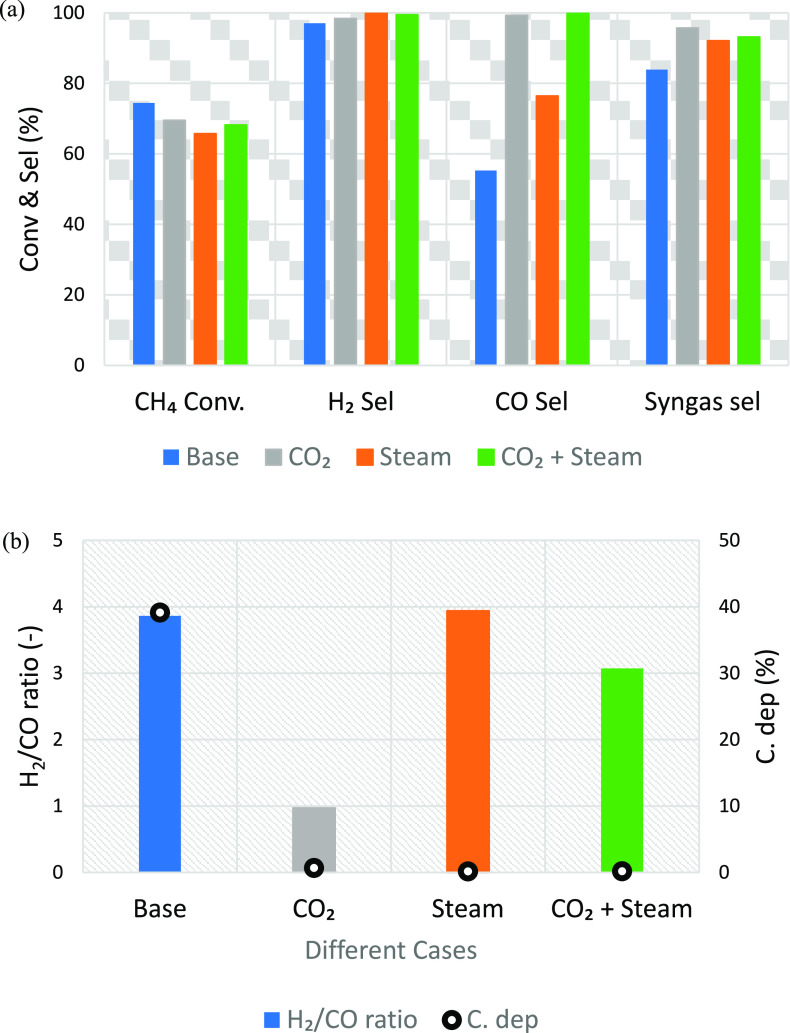
(a) Effect of steam and CO_2_ utilization at the fuel stage on fuel conversion and selectivity and (b) the effect of steam and CO utilization at the fuel stage on syngas quality (H_2_/CO ratio) and carbon deposition at 50% CH_4_ molar fraction, 950 °C, and 1 bar. (i) Fuel stage, base case (gas input: CH_4_, 4.1 nL/min; N_2_, 4.1 nL/min for 2.93 min); CO_2_ case (gas input: CH_4_, 4.1 nL/min; CO_2_, 4.1 nL/min for 2.93 min); CO_2_+H_2_O case (gas input: CH_4_, 4.1 nL/min; CO_2_, 2.05 nL/min; H_2_O, 2.05 nL/min for 2.93 min); H_2_O case (gas input: CH_4_, 4.1 nL/min; H_2_O, 4.1 nL/min for 2.93 min); (ii) N_2_ purge (gas input: N_2_, 10 nL/min for 5 min); (iii) steam stage (gas input: H_2_O, 2 nL/min for 10 min); (iv) air stage (gas input: air, 10 nL/min for 3 min).

The CO_2_ case shows that carbon deposition was reduced significantly (from 40% to 0%) with a resultant improvement in the purity of the H_2_ produced in the subsequent steam stage (Figure 11). A similar approach was applied in chemical looping reforming using a perovskite-based oxygen carrier where a combined effect of POX (reaction R3) and DMR (reaction R4) was achieved.^1,70^ The attractiveness of this strategy is the ability to utilize CO_2_ to produce valuable products and offset GHG emissions. The H_2_O case was considered to achieve a combined effect of POX (reaction R3) and SMR (reaction R5). With this arrangement, carbon deposition was significantly reduced achieving a high H_2_/CO ratio, which was found to be close to 4 (Figure 12b) due to the WGS reaction (reaction R11). The converted steam reacted with the produced CO to form CO_2_ (and H_2_) through the aforementioned WGS reaction (evidenced by the presence ofCO_2_ as a product in the fuel stage as shown in Figure 11). 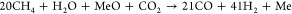
R13It is also possible to synergize CO_2_ and H_2_O utilization in the fuel stage to achieve a combined effect of POX, DMR, and SMR (reaction R13), known as trimethane reforming (TMR). TMR is expected to eliminate the disadvantages of the conventional individual reactions, improve overall process performance, efficiency, prolong catalyst life, and mitigate coking.^3,71^ TMR also provides the flexibility to tune the produced syngas to a desired quality. This approach has been previously demonstrated to produce syngas with a H_2_/CO ratio between 1 and 2, suitable for gas-to-liquid processes.^71^

It was observed that the three cases with the addition of an oxidant (H_2_O and/or CO_2_), syngas production was favored from the start of the fuel step, thus eliminating the initial reduction of the oxygen carrier that produced CO_2_ and steam. This has resulted in a slight decrease in the overall methane conversion for those three cases as can be seen in Figure 12a. On the other hand, the overall H_2_, CO, and syngas selectivities improved compared with the base case (Figure 12a).

The slight improvement in H_2_ selectivity resulted from the disappearance of the reduction step at the beginning of the fuel stage which eliminates steam production that affects H_2_ selectivity. Instead, methane was reformed to syngas (H_2_ + CO) in the presence of the oxidant. Carbon deposition decreased substantially in the presence of the oxidant, thus considerably improving the CO selectivity (Figure 12b). The improvement in CO selectivity was however lower for the case of pure steam addition, which could be attributed to the occurrence of the WGS reaction in the presence of steam thus maximizing hydrogen production. With these results, the CO_2_ and the CO_2_ + steam cases could safely be recommended for GTL applications due to the moderate H_2_/CO ratio, the elimination of carbon deposition with high syngas selectivity, but interestingly, they can also produce high purity H_2_ in the steam stage.

**Figure 13:**
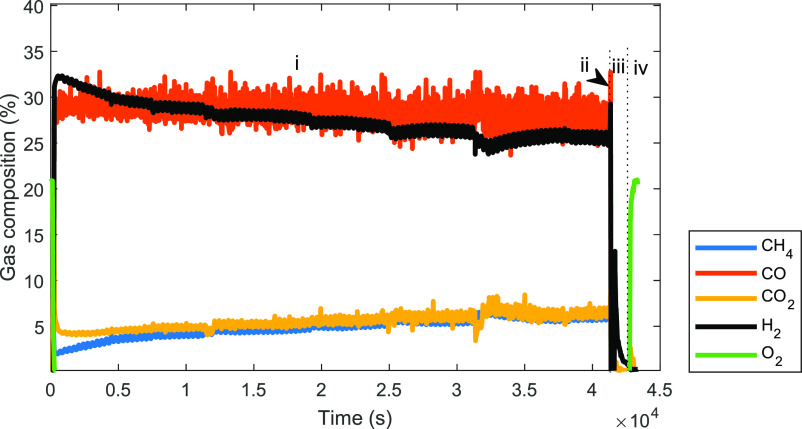
Transient gas composition of GSPOX after 12 h at CH_4_ molar fraction of 50% in the fuel stage (CO_2_/CH_4_ ratio = 1:1) at 1 bar, and 950 °C. (i) fuel stage (gas input: CH_4_, 4.1 nL/min; CO_2_, 4.1 nL/min for 12 h); (ii) N_2_ purge (gas input: N_2_, 10 nL/min for 5 min); (iii) steam stage (gas input: H_2_O, 2 nL/min for 10 min); (iv) air stage (gas input: air, 10 nL/min for 3 min).

The improvement of Figure 12 in the fuel stage when cofeeding an oxidant with methane could be attributed to two mechanisms: (i) simultaneous redox reactions occur in the presence of the oxidant leading to the immediate restoration of the lattice oxygen in the reduced perovskite,^53,72^ (ii) oxidant addition could also ensure simultaneous gasification of the deposited carbon to CO thus eliminating its negative effect on syngas quality (H_2_/CO ratio). An additional experiment was performed by cofeeding CO_2_ and CH_4_ (50% molar fractions each) for more than 12 h (Figure 13), which demonstrated that syngas production could be sustained continuously with only a very small drop (<5%) in the conversion of CH_4_. This indicates that the oxygen carrier performed similarly to a catalyst in the dry reforming reaction. At the start of the fuel stage, CH_4_ conversion was slightly higher than CO_2_ conversion but gradually decreased and stabilized at the same value as the CO_2_ conversion for the rest of the stage (the CO_2_ conversion remained constant in the entire duration of the fuel stage).

From an XRD measurement (Figure 6a) of the oxygen carrier sample collected immediately after the fuel stage (before the reoxidation step), it is evident that the oxygen carrier was not reduced significantly when CH_4_ and CO_2_ were cofed. The small shift in peak position toward lower diffraction angles indicates that only a small amount of lattice oxygen was removed (∼0.4 wt %), most likely at the beginning of the experiment shown in Figure 13. It was observed that a ratio of CH_4_/CO_2_ > 3 was required to reduce the oxygen carrier further and utilize its complete oxygen storage capacity of ∼9 wt %. Below that ratio, the oxygen carrier maintained its high oxidation state without undergoing a bulk phase transition; however, full recovery of its lattice oxygen required a stronger oxidant, that is, air (reaction R12). Therefore, the observations made do not suggest the catalytic activation of CH_4_ or CO_2_ that is mechanistically comparable with the conventional dry reforming of methane, since the perovskite itself is not catalytically active. The trend seen in Figure 13 appears to be rather the result of the simultaneous reduction/oxidation of the oxygen carrier utilizing only a small amount of its lattice oxygen. However, further investigations under kinetically controlled conditions are required to fully decipher and understand the nature of these observations.

At the beginning of the fuel stage, the rate of reduction of the oxygen carrier to H_2_ was higher than the rate of oxidation but gradually decreased and remained constant following the same trend as CH_4_ conversion later in the stage. Altogether Figure 13 suggests that syngas production was likely following the aforementioned mechanism (i) exposing the oxygen carrier to simultaneous reduction through partial oxidation by CH_4_ and oxidation by CO_2_. At the end of the 12 h fuel stage, only 4% degree of reduction of the oxygen carrier was achieved similar to the degree of reduction achieved after 3 min of the fuel stage with the same H_2_ yield at the subsequent steam stage. This suggests that when cofeeding an oxidant with CH_4_ into this oxygen carrier, simultaneous redox reactions (oxidation and reduction) can take place at equal rates, when the oxygen carrier is reduced to 4%, as observed in Figure 13. Again, further research is needed for drawing firm conclusions about the mechanisms by which syngas is produced when cofeeding an oxidant with CH_4_ to the oxygen carrier.

### 3.3 The Effect of Pressure

**Figure 14:**
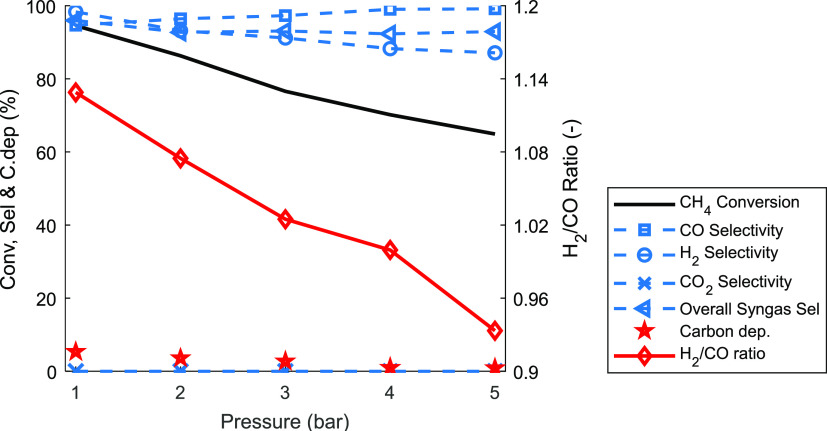
Variation of gas composition with pressure at 50% CH_4_ molar fraction and 950 °C. (i) Fuel stage (gas input: CH_4_, 2.1–10.5 nL/min; CO_2_, 2.1–10.5 nL/min for 11.74–2.35 min); (ii) N_2_ purge (gas input: N_2_ 10–50 nL/min for 10–2 min); (iii) steam stage (gas input: H_2_O, 2–10 nL/min for 20–4 min); (iv) air stage (gas input: air, 10–50 nL/min for 10–2 min).

Pressurized operation is necessary to reduce downstream compression work, improve process efficiency, and explore the feasibility of integration with other downstream processes. For these reasons, a further investigation of GSPOX at pressures from 1–5 bar was performed at 50% CH_4_ molar fraction, the addition of CO_2_ (CO_2_/CH_4_ ratio of 1), and an operating temperature of 950 °C. The gas feed was increased proportionally to the pressure to maintain a constant superficial gas velocity of about 0.1 m/s in the reactor. The achieved performance is summarized in Figure 14. It can be seen that increasing the pressure led to a decrease in CH_4_ and CO_2_ conversions, similar to a previous study.^73^ Since the reactions are heterogeneous (gas/solid reaction) and mainly endothermic, it is possible that the pressure would have negative effects both on the equilibrium and the reaction kinetics. The overall CO_2_ conversion was lower than the CH_4_ conversion, confirming that the partial oxidation of CH_4_ occurs at a faster rate than the oxidation of the metal oxide by CO_2_ at the beginning of the fuel stage as shown earlier in Figure 13. However, the difference between the two reactions was found to decrease with the pressure, indicating that CH_4_ conversion is affected more negatively by pressure than CO_2_ conversion. This could be attributed to the fact that CH_4_ is involved in more reaction pathways (reaction R3, reaction R4, reaction R5, and reaction R7) while CO_2_ is involved in fewer reactions (reaction R4 and reaction R6). The decrease in H_2_ selectivity indicates that pressure improves the kinetics of the RWGS reaction (reaction R6) in which CO_2_ reacts with the H_2_ to form H_2_O and CO indicating that kinetics played a larger role than thermodynamics. This leads to a decrease of the syngas H_2_/CO ratio with pressure (even below 1 at pressures higher than 4 bar). Overall, further work is needed to optimize this oxygen carrier to minimize the negative effect of pressure on its performance before the scale-up of the GSPOX process.

## 4 Conclusion

The coupling of CH_4_ partial oxidation and water splitting for syngas and hydrogen production as an efficient pathway for natural gas decarbonization was investigated in this work using a lanthanum strontium ferrite oxygen carrier. Unlike previous studies on related topics, the experiments were completed in a novel chemical looping reactor concept known as gas switching technology (GST) that uses a single fluidized bed reactor cycling multiple stages of the process (fuel, steam, and air stages). The results showed that the oxygen carrier exhibits high selectivity to syngas production at the fuel stage but with substantial carbon deposition when pure methane was fed, resulting in syngas production with a very high H_2_/CO ratio in the fuel stage and very low purity H_2_ production in the consecutive steam stage. If only syngas is targeted, carbon deposition will not be problematic as the deposited carbon could totally be gasified in the steam stage producing valuable syngas and ensuring complete regeneration of the oxygen carrier, thus prolonging its lifetime with sustained chemical reactivity.

Co-feeding an oxidant, such as CO_2_, H_2_O, or both, together with CH_4_ at the fuel stage resulted in a significant decrease in carbon deposition and the H_2_/CO ratio between 1 and 4. This demonstrates an important feature of GSPOX, which is the tunability of syngas composition to properly respond to the needs of the different GTL downstream processes. For all cases of H_2_O and CO_2_ (or combination) utilization at the fuel stage, an improved H_2_ purity at the steam stage was achieved following the reduction in carbon deposition with less CO contamination through the gasification of the deposited carbon with H_2_O.

An important observation of continuous syngas production with (H_2_/CO ≈ 1) by cofeeding CO_2_ and CH_4_ at the fuel stage for over 12 h indicated that the oxygen carrier was exposed to simultaneous redox reactions through CH_4_ partial oxidation with the lattice oxygen which is restored instantly by the fed CO_2_. This process occurs at a higher rate for the CH_4_ partial oxidation in the beginning of the fuel stage but reduces gradually to equalize the reversed oxidation reaction by CO_2_ resulting in a behavior similar to conventional methane reforming that occurs continuously as long as heat is supplied.

Operating at high pressures was found to have negative effects on both CH_4_ and CO_2_ conversions. This could be due to the combined equilibrium and kinetic limitations of the involved endothermic heterogeneous reactions. CO_2_ conversion was less sensitive to the pressure than CH_4_ conversion since CH_4_ is involved in more dominating reaction pathways than CO_2_. Pressure improves the kinetics of the RWGS reaction contrarily to equilibrium prediction, thus affecting the H_2_ selectivity and the syngas H_2_/CO ratio negatively. This calls for further research to explore approaches to minimize the negative impact of the pressure on the GSPOX performance before scale-up. A dedicated techno-economic assessment is also recommended to confirm the GSPOX attractiveness against benchmarking technologies.

## Abbreviations

ASU: air separation unit
ATR: autothermal reforming
CAPEX: capital expenditure
CCUS: carbon capture utilization and storage
CFB: circulating fluidized bed
CLPOX: chemical looping partial oxidation
CLR: chemical looping reforming
CMR: combined methane reforming
DMR: dry methane reforming
GSPOX: gas switching partial oxidation
GSR: gas switching reforming
GST: gas switching technology
GTL: gas-to-liquid
Me: metal
MeO: metal oxide
OC: oxygen carrier
POX: partial oxidation
RWGS: reverse water gas shift
SMR: steam methane reforming
TMR: trireforming
*U*: fluidization velocity
*U*_mf_: minimum fluidization velocity
WGS: water gas shift
XRD: X-ray diffraction
**Symbols:**
*C*_dep_: carbon deposition
*D*_10_: diameter of the catalyst which 10% of a sample mass is smaller than
*D*_50_: diameter of the catalyst which 50% of a sample mass is smaller than
*D*_90_: diameter of the catalyst which 90% of a sample mass is smaller than
*n*_C__,out_fuel_: mole of C at the gas outlet during reforming stage
*n*_CH_4_in_fuel_: mole of CH_4_ fed in the fuel stage
*n*_CH_4_,out_fuel_: mole of CH_4_ at the gas outlet in the fuel stage
*n*_*CO*__,*out*_*oxi*_: mole of CO at the gas outlet in the oxidation stage
*nCO*_2__,out_oxi_: mole of CO_2_ at the gas outlet in the oxidation stage
*n*_CO__,out_fuel_: mole of CO at the gas outlet in the fuel stage
*n*_CO_2_,in_fuel_: mole of CO_2_ fed in the fuel stage
*n*_CO_2_,out_fuel_: mole of CO_2_ at the gas outlet in the fuel stage
*n*_H_2_,__out_fuel_,: mole of H_2_ at the gas outlet in the fuel stage
*n*_H_2_O,out_fuel_: mole of H_2_O at the gas outlet in the fuel stage
*n*_*i*__out_: mole of any gas species at the gas outlet
*n*_N_2_,in_: known mole of N_2_ gas fed in the fuel stage
*S*_CO_: CO selectivity
*S*_CO_2__: CO_2_ selectivity
*S*_H_2__: H_2_ selectivity
*S*_syngas_: overall syngas selectivity
*x*_*i*__out_: mole fraction of any gas species as recorded in the gas analyzer
*x*_N_2_out_: mole fraction of N_2_ gas as recorded in the gas analyzer
γ_CH_4__: CH_4_ conversion
γ_CO_2__: CO_2_ conversion
*γ*_syngas_: syngas yield
